# Multilocus phylogenetic analyses reveal unexpected abundant diversity and significant disjunct distribution pattern of the Hedgehog Mushrooms (*Hydnum* L.)

**DOI:** 10.1038/srep25586

**Published:** 2016-05-06

**Authors:** Bang Feng, Xiang-Hua Wang, David Ratkowsky, Genevieve Gates, Su See Lee, Tine Grebenc, Zhu L. Yang

**Affiliations:** 1 Key Laboratory for Plant Diversity and Biogeography of East Asia, Kunming Institute of Botany, Chinese Academy of Sciences, Kunming, China; 2 Tasmanian Institute of Agriculture, and School of Plant Science, University of Tasmania, Hobart, Tasmania, Australia; 3 Tanarimba, Janda Baik, Bentong 28750 Bentong, Pahang, Malaysia; 4 Slovenian Forestry Institute, Večna pot 2, SI-1000 Ljubljana, Slovenia

## Abstract

*Hydnum* is a fungal genus proposed by Linnaeus in the early time of modern taxonomy. It contains several ectomycorrhizal species which are commonly consumed worldwide. However, *Hydnum* is one of the most understudied fungal genera, especially from a molecular phylogenetic view. In this study, we extensively gathered specimens of *Hydnum* from Asia, Europe, America and Australasia, and analyzed them by using sequences of four gene fragments (ITS, nrLSU, *tef*1α and *rpb*1). Our phylogenetic analyses recognized at least 31 phylogenetic species within *Hydnum*, 15 of which were reported for the first time. Most Australasian species were recognized as strongly divergent old relics, but recent migration between Australasia and the Northern Hemisphere was also detected. Within the Northern Hemisphere, frequent historical biota exchanges between the Old World and the New World via both the North Atlantic Land Bridge and the Bering Land Bridge could be elucidated. Our study also revealed that most *Hydnum* species found in subalpine areas of the Hengduan Mountains in southwestern China occur in northeastern/northern China and Europe, indicating that the composition of the mycobiota in the Hengduan Mountains reigion is more complicated than what we have known before.

*Hydnum* L. is a fungal genus with considerable ecological and economic importance as it comprises of several edible ectomycorrhizal mushrooms. Species of this genus have been reported from North/Central America^1^,^2^,^3^, Europe^4^, East Asia^5^,^6^,^7^, Australasia^8^,^9^,^10^, and sporadically from Africa^11^ and South America^12^. Different species of *Hydnum* can be found under plants from several families, such as Pinaceae, Fagaceae, Dipterocarpaceae and Myrtaceae. Previous studies have shown that they can form ectomycorrhizal relationships with these plant families^8^,^13^,^14^ and thus are very important for the maintenance of forest ecosystems. Due to their edibility, mushrooms of *Hydnum* are consumed and traded in markets in many countries all over the world, such as China, the USA, Mexico and Spain^15^,^16^.

The genus name *Hydnum* was proposed by Linnaeus at the dawn of modern taxonomy^17^. Originally it was used to infer mushrooms with aculeate (tooth-like) hymenophores but has later been restricted to those with a fleshy fruiting body and colorless smooth spores^18^. According to molecular evidence, this genus belongs to Hydnaceae, Cantharellales^19^,^20^. Since the establishment of this genus, studies on *Hydnum* have been regional and mainly morphology-based for over 250 years. The most used macro- and micro-morphological characters include the size and color of fruiting bodies, as well as the shape and size of basidiospores. During this period, several species were described from different continents, but only seven of them have been widely accepted^18^,^21^,^22^. They are *H. repandum* L. and *H. rufescens* Pers. from Europe, *H. albidum* Peck, *H. albomagnum* Banker and *H. umbilicatum* Peck from North America^1^,^2^,^3^, *H. crocidens* Cooke and *H. elatum* Massee from Australasia^9^,^10^. With the aid of sequences of the internal transcribed spacer (ITS) and careful microscopic observations, European mycologists described one new species, *H. ellipsosporum* Ostrow & Beenken, from the *H. rufescens* species complex in 2004^23^. Since then, over ten phylogenetic species within the species complexes of *H. rufescens* and *H. repandum* have been identified by analyzing the divergences of ITS sequences of European samples^4^. Three of them have later been formally described as *H. magnorufescens* Vizzini *et al.*^24^, *H. vesterholtii* Olariaga *et al.* and *H. ovoideisporum* Olariaga *et al.*^21^, respectively. Recently, a new species with whitish basidiomata, *H. minum* Yanaga & N. Maek., was described from Japan^22^. These studies strongly suggest that the species diversity of *Hydnum* had been heavily under-estimated due to the lack of molecular evidence. Unfortunately, few molecular phylogenetic studies have been conducted on *Hydnum* collections made from continents outside of Europe.

Although many regional morphological studies have been made on *Hydnum*, especially in North America, Europe and Australia, the distribution pattern of this genus is still not clear. Most species within this genus, like *H. albomagnum*, *H. elatum* and *H. umbilicatum*, were reported as endemic to certain continents, while some other species, such as *H. albidum*, *H. repandum* and *H. rufescens*, were assumed to be holarctic species disjunctly distributed in North America, Europe and East Asia. *Hydnum crocidens,* which was originally described from Australia, was once considered to be “unseparable from *H. repandum*”^18^, but controversial opinions also existed^8^. Given the fact that the species complexes of *H. rufescens* and *H. repandum* can be split into several separate species by using samples collected merely from southern Europe^4^,^21^,^23^,^24^, one can hypothesize that the traditional view on the distribution pattern of *Hydnum* should be largely revised with the add-in of molecular analyses based on an extensive sampling through its distribution range.

Biogeographic analysis is one important way to understand how species have formed and evolved. Many biogeographic studies have been carried out on different fungal groups in recent years, several of which have pointed out that paleotropical areas could have acted as the cradle for some fungal groups, such as the porcini mushrooms^25^ and some sections of *Amanita*^26^,^27^. *Hydnum* species are known from temperate and tropical regions of both the Northern and Southern Hemispheres. Whether it has experienced a similar evolutionary history like the fungal groups mentioned previously is still an open question.

The objective of this study is to elucidate the species diversity and the biogeography of *Hydnum* by using multilocus phylogenetic analyses on collections made from the currently known distribution range of this fungal genus. Special attention was given to the following regions: (i) East Asia. Both morphological and molecular studies on *Hydnum* are currently severely lacking in this region, but tremendous morphological and ecological diversities have been observed in our earlier survey of this genus. Meanwhile, several studies have indicated that the fungal diversity in this region has been heavily under-estimated; (ii) North America and Australasia. There is currently no reported molecular study on samples collected from these two regions. Furthermore, species from Australasia have their own unique host plants (Dipterocarpaceae and Myrtaceae). We try to answer the following two specific questions: (i) How many species of *Hydnum* can be identified from a molecular phylogenetic view? (ii) Is there a certain distribution pattern (endemism or disjunction) for *Hydnum* worldwide, and, if yes, how has this distribution pattern been formed?

## Results

### Phylogenetics

**Figure 1:**
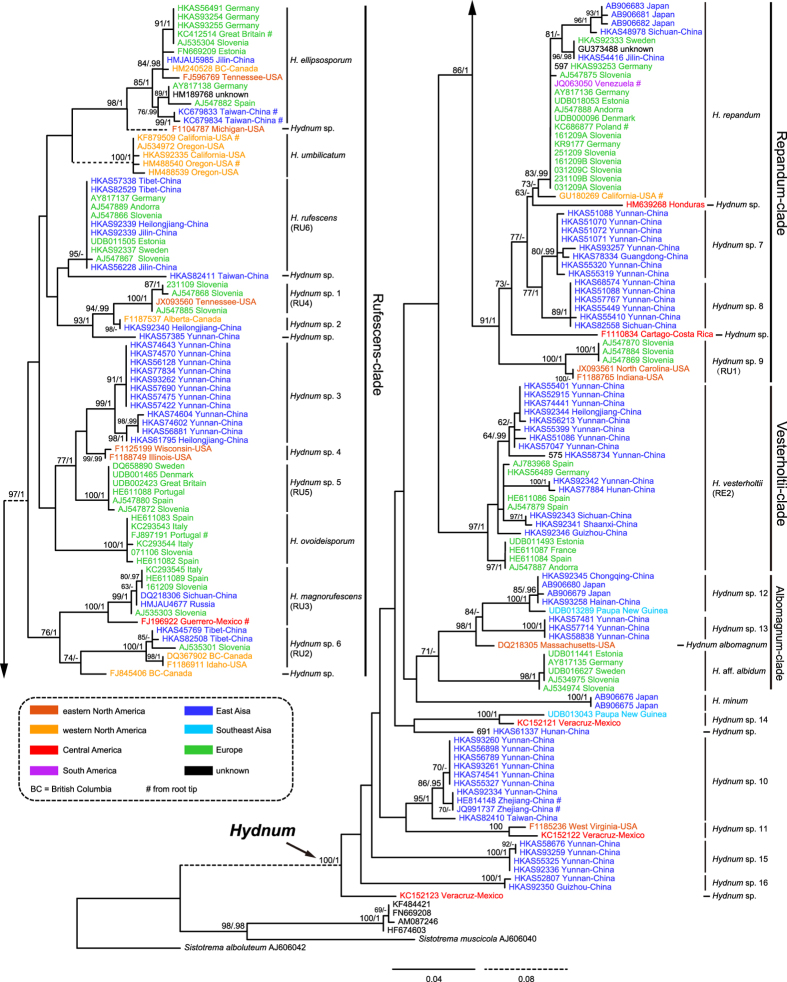
Phylogenetic tree inferred from the Maximum Likelihood (ML) analysis based on the ITS dataset I. Bootstrap values (ML)/posterior probabilities (from Bayesian Inference) are shown above or beneath individual branches. Only bootstrap values larger than 60 and posterior possibilities over 0.95 are shown.

**Figure 2:**
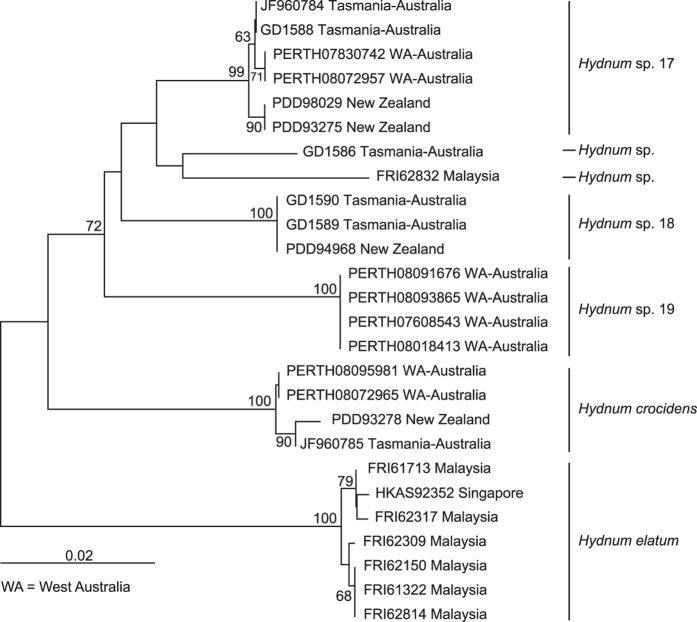
Phylogenetic tree inferred from the Neighbor-Joining (NJ) analysis based on the ITS dataset II. Bootstrap values are shown above individual branches. Only values larger than 60 are shown.

**Figure 3:**
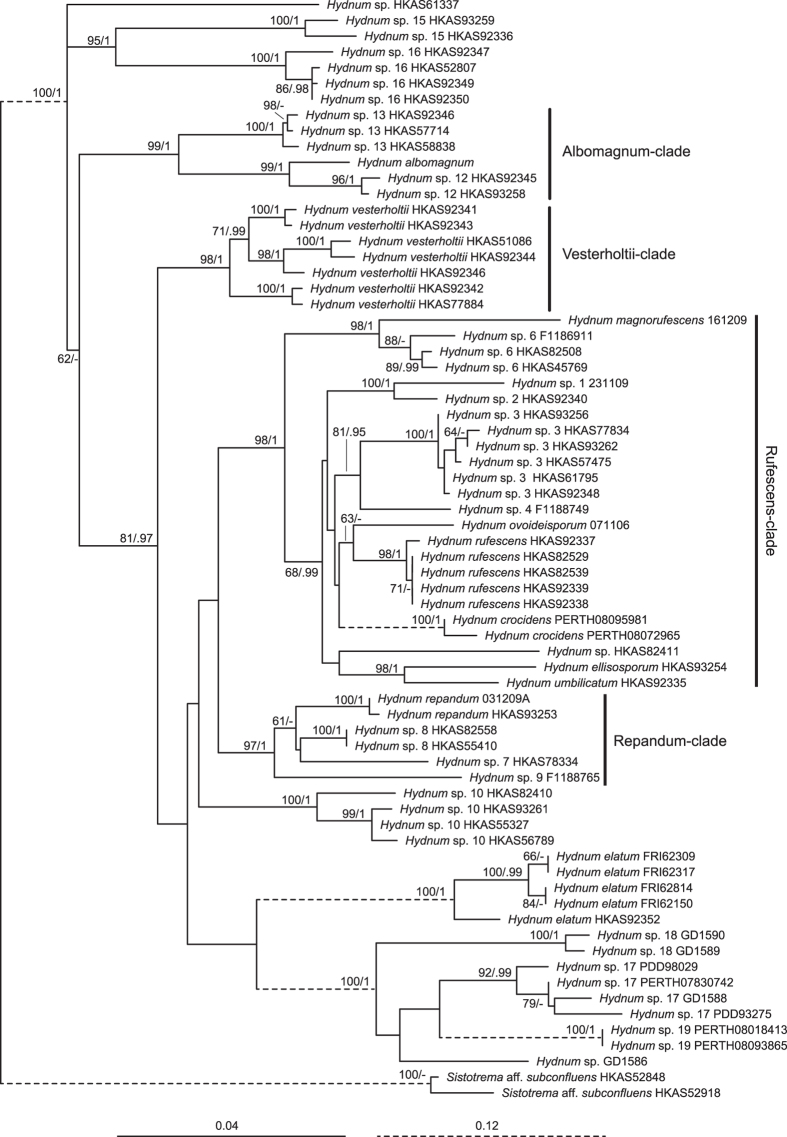
Phylogenetic tree inferred from the Maximum Likelihood (ML) analysis based on the three-gene dataset of nrLSU, *rpb*1 and *tef*1*α*. Bootstrap values (ML)/posterior probabilities (from Bayesian Inference) are shown above or beneath individual branches. Only bootstrap values larger than 60 and posterior probabilities over 0.95 are shown.

The ITS dataset I included 188 sequences (six as outgroups), 98 of which were newly generated in this study. Each entry contained 701 aligned bases, with 251 bases for ITS1, 154 for 5.8 S, and 296 for ITS2. Maximum Likelihood (ML) and Bayesian Inference (BI) analyses yielded identical tree topologies and thus only the tree inferred from the ML analysis is shown (Fig. 1). Four well-supported lineages, named here as *Albomagnum*-clade, *Repandum*-clade, *Rufescens*-clade and *Versterholtii*-clade, could be recovered by using this dataset, but their relationships could not be well elucidated. Meanwhile, no obvious basal group for *Hydnum* could be identified. The ITS dataset II included 26 sequences, 24 of which were newly added in this study. The nrLSU, *rpb*1 and *tef*1α matrices harbored 56, 60 and 57 sequences, respectively, of which 55, 60 and 56 sequences were newly generated. The phylogenetic trees inferred from these four matrices are shown in Fig. 2 and Supplementary Figs S1–3. The three-gene dataset contained sequences from 70 specimens. Each of them contained 2610 aligned sites, with 1308, 870 and 429 sites for nrLSU, *rpb*1 and *tef*1α, respectively. ML and BI analyses yielded similar topologies and only the tree constructed by the ML strategy is shown in Fig. 3. The three-gene dataset inferred the four lineages identified by the ITS dataset I but also failed to clearly address their relationships. Similar to the result inferred from ITS dataset I, no obvious basal group could be detected. *Hydnum crocidens* from Australia and New Zealand clustered within the *Rufescens*-clade. The remaining Australasian samples formed two separate clades, one represented by *H. elatum* collected from Singapore and Malaysia, and the other represented by samples from Australia and New Zealand. These two clades are strongly divergent from each other and also from the samples gathered from the Northern Hemisphere.

### Phylogenetic species delineation

The ITS dataset I and II identified 26 and five monophyletic clades respectively. (Figs 1 and 2). Among them, 12 and four clades, respectively, could be assigned to formally described species (with Latin names) and some undescribed phylogenetic species inferred by a previous study^4^ (*Hydnum* spp. 1, 5, 6 and 9). Among the remaining 15 clades, 11 clades (*Hydnum* spp. 3, 6, 8, 10, 12, 13, 15–19) were well-supported by at least two of the nrLSU, *rpb*1 and *tef*1*α* matrices (Supplementary Figs S1–3). Therefore, each of them could be defined as a phylogenetic species. Unfortunately, we failed to obtain sequences of nrLSU, *rpb*1 and *tef*1*α* from several samples of *Hydnum* spp. 1, 5, 6 and 9. Each of them was provisionally accepted as a phylogenetic species.

### Molecular dating

**Figure 4:**
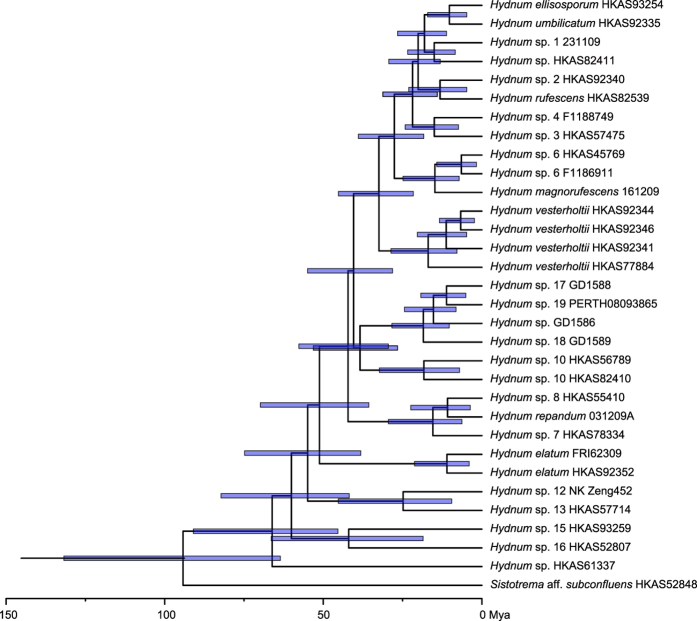
The divergence time of *Hydnum* species estimated by using BEAST. The pale blue bars show the 95% highest posterior density (HPD) for the divergence time of each node.

The best molecular clock model selected for the divergence time estimation was the relaxed exponential clock model. The chronogram generated by using this model is provided in Fig. 4. Our analysis estimated that *Hydnum* originated ca. 67 million years ago (Mya, 95% HPD = 45.38–90.92 Mya).

## Discussion

Previous regional morphology-based taxonomical studies on *Hydnum* have largely underestimated the true species diversity of this genus due to the morphological paucity, the lack of a worldwide survey of *Hydnum* resources and the lack of molecular evidence. Our multilocus phylogenetic analyses on worldwide *Hydnum* samples provide some new insights into the cloudy species diversity of this genus. Prior to this study, a total of 12 species of *Hydnum* have been formally described^4^,^18^,^21^,^22^,^23^,^24^, 11 of which were included in our phylogenetic analyses. The only exception is *H. albidum*, a species originally described from North America. Currently, some European samples (Fig. 1, marked as “*H.* aff. *albidum*”) are named as *H. albidum.* However, we cannot determine if this treatment is reasonable or not unless samples of *H. albidum* from North America are available for phylogenetic analysis. Along with these species, four additional phylogenetic species have been previously identified from Europe by the evidence of ITS sequences^4^. Our phylogenetic analyses revealed at least 31 phylogenetic species (each represented by at least two collections) within *Hydnum*, 15 of which (about 50%) are uncovered for the first time (Figs 1 and 2). These newly identified species were mainly collected from North/Central America, East Asia and Australasia. In addition, we found ten putative phylogenetic species which harbor only one collection each (Figs 1 and 2, named as *Hydnum* sp.). They are not included in the following discussion.

Five species, *H. albidum*, *H. albomagnatum*, *H. umbilicatum, H. repandum* and *H. rufescens*, have been recorded from North/Central America, with the former three species originally described from this region. We failed to find *H. repandum* and *H. rufescens* in currently available specimens from this region. However, evidence from root tips of *Pinus muricata* (Fig. 1, GU180269) indicated that *H. repandum* occurs in western North America. From this study, we identified at least nine additional (phylogenetic) species from this continent. They are *H. ellipsosporum*, *H. magnorufesecens*, *Hydum* spp. 1–2, 4, 6, 9, 11 and 14. Most of these species were clustered into the *Rufescens-*, *Repandum-* and *Albomagnum-*clades, but the latter two phylogenetic species each formed a single clade without any sister group clearly identified (Fig. 1).

Three species, *H. albidum*, *H. repandum* and *H. rufescens*, have been recorded from East Asia without any molecular evidence^6^,^7^,^28^,^29^. A new species, *H. minum*, has been recently described from Japan with molecular and morphological evidence^22^. Additionally, *H. magnorufescens*, a species originally described from Europe, has been shown by molecular evidence to occur in Southwest China^24^. As no representative of *H. albidum* was included in our study, we are not sure whether this species occurs in East Asia or not. *Hydnum repandum* and *H. rufescens* were shown to be distributed in this region by many specimens collected by ourselves. Besides, we revealed 11 additional (phylogenetic) species from East Asia. They are *H. ellipsosporum*, *H. vesterholtii*, *Hydnum* spp. 2, 3, 6–8, 10, 12–13, and 15–16. These species were clustered into the *Rufescens-*, *Repandum-*, *Vesterholtii-* and *Albomagnum-*clades, except that *Hydnum* spp. 10, 15 and 16 remained unassigned (Fig. 1).

Two well-accepted species, *H. elatum* and *H. crocidens*, were originally described from Australasia. Our phylogenetic analyses confirmed the independence of these two species. Meanwhile, we uncovered at least five new phylogenetic species from this region. Two species represented by samples collected from Papua New Guinea (*Hydnum* spp. 12 and 14) clustered together with samples from the Northern Hemisphere, while the other three species gathered from Australia and New Zealand (*Hydnum* spp. 17–19) formed two distinct clades (Figs 1 and 2).

**Figure 5:**
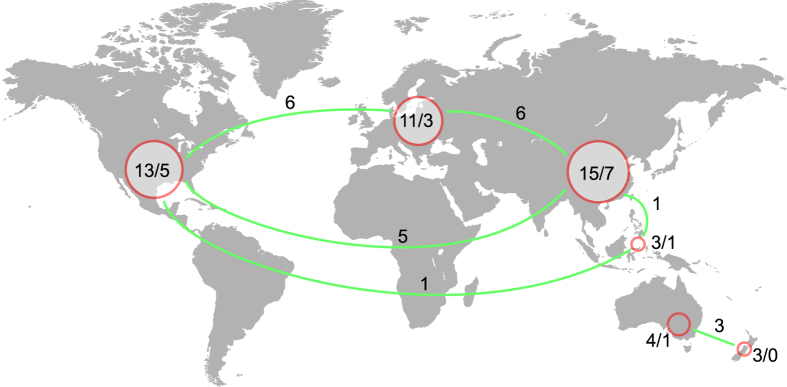
A map showing the disjunct distribution of *Hydnum* species. Cycles show different continents, while the numbers in or near each of these cycles indicate the total/endemic (phylogenetic) species in certain continents. The numbers above the green lines reveal the number of species shared by pairs of continents. The map was generated using the free application maptools v0.8–39 (http://CRAN.R-project.org/package=maptools) in the R environment and then modified in Adobe Illustrator CS6 (Adobe Systems Inc., CA, USA).

In summary, a total of 15, 13 and seven (phylogenetic) species of *Hydnum* can be found in East Asia, North/Central America and Australasia, respectively. We did not identify any additional phylogenetic species from Europe, a region where 11 (phylogenetic) species had been recorded in previous studies. The relatively even distribution of *Hydnum* species in different continents of the Northern Hemisphere is mainly due to the existence of several disjunctly distributed species (Figs 1 and 5), which could be attributed to frequent historical fungal exchanges in this region (see discussion below).

Although an extensive study of *Hydnum* has been made here, our multilocus phylogenetic analyses failed to infer a clear phylogenetic relationship among the major clades within *Hydnum*. The lack of backbone supports could be explained from two aspects: (i) the survey and sampling for *Hydnum* from Africa and its adjacent tropical regions, like Madagascar and Seychelles, as well as South America, are still lacking; (ii) several ancestral species of modern taxa could have experienced extinction events, which makes it impossible to clearly reconstruct the phylogenetic relationships of modern species. Despite this, some new insights into the historical biogeography of *Hydnum* could be inferred from currently available data.

Australasian samples analyzed in this study could be divided into seven phylogenetic species based on ITS sequences (Figs 1 and 2). Five of these species, *H. crocidens*, *H. elatum* and *Hydnum* spp. 17–19, showed strong ITS sequence divergences from species collected from the North Temperate Zone, which made it impossible to analyze them together in a single ITS dataset. In our phylogenetic analyses based on the three-gene dataset of nrLSU, *tef*1α and *rpb*1, only *H. crocidens* was clustered into the *Rufescens*-clade (with a long branch length and no clear sister group inferred); the remaining four species formed two clades significantly divergent from other clades made up by samples from the Northern Hemisphere. All of these observations strongly indicate that these species could be old relics held in this region for a long historical period. These species can form putative symbiotic relationships with plants of Dipterocarpaceae and Myrtaceae, which are indigenous to pantropical areas. Ecological adaptation to certain host plants could also have accelerated their divergences from other species. Interestingly, no species was detected to be shared by Southeast Asia and Australia/New Zealand. In contrast, all three species from New Zealand distinguished in this study are conspecific with those from southwestern and southern Australia (West Australia and Tasmania) (Figs 2 and 5), indicating recent biota exchanges of *Hydnum* between these two regions. This could have been introduced by a co-migration of plants with their fungal partners, as the putative host plants of these species, *Leptospermum*^30^, has recently migrated to New Zealand from Australia^31^,^32^.

Two *Castanopsis*-associated collections made from Papua New Guinea (under ITS accession numbers UDB013289 and UDB013043), were revealed to be phylogenetically close to tropical/subtropical East Asian and neotropical Mexican samples, respectively (Fig. 1). UDB013289 is nearly identical to samples from Chongqing and Hainan, China, indicating a recent migration event between East Asia and Southeast Asia. Although the direction of migration could not be inferred using our data, we hypothesize that it should be from East Asia to Southeast Asia as suggested by the migratory route of *Castanopsis*^33^. The intercontinental species from Mexico (KC152121) and Papua New Guinea (UDB013043) could be explained from two alternative aspects, a long distance dispersal by spores or a step by step migration between Central America and Papua New Guinea by using North America and East Asia as corridors. However, the ectomycorrhizal nutritional mode of *Hydnum,* like its relatives *Cantharellus* and *Clavulina*, would strongly decrease the ratio of successful establishment by long distance dispersal of spores^34^. Thus, a step by step migration could be more reasonable to explain the disjunction between Central America and Southeast Asia. In fact, we did find some clues that East Asia shares some species with Central America, like *H. magnorufescens* and *H. repandum* (also see discussion below).

For the samples collected from the North Temperate Zone, we found strong evidence of disjunct distributions of *Hydnum* between the New World and the Old World (Figs 1 and 5). Prior to this study, several biogeographical studies have indicated significant endemism in both symbiotic (like the porcini mushrooms and lethal *Amanita* species) and saprophytic (like *Singerocybe* and *Heterobasidion*) fungi^25^,^26^,^35^,^36^. Contrary to these findings, we illustrated here nine intercontinental species within *Hydnum* in the Northern Hemisphere (Fig. 1). Four kinds of disjunct or discontinuous distribution could be inferred from this fungal genus: (i) holarctic distribution represented by *H. repandum*, *H. ellipsosporum*, *H. magnorufescens* and *Hydnum* sp. 6; (ii) North/Central American–European disjunction found in *Hydnum* spp. 1 and 9; (iii) North/Central American–East Asian disjunction found in *Hydnum* sp. 2; (iv) East Asian–European discontinuous distribution represented by *H. rufescens* and *H. vesterholtii*. Besides, we could identify several intercontinental species pairs in different major clades, such as *Hydnum* spp. 3–5 and species within the *Repandum*- and *Albomagnum-*clades (Fig. 1).

The abundant intercontinental species and species pairs would indicate frequent historical biota exchanges within *Hydnum* between the Palaeoarctic and the Neoarctic. Land bridges have been widely accepted as the corridors for such biota exchanges between these two regions in previous biogeographic studies on plants and ectomycorrhizal fungi^25^,^26^,^37^,^38^,^39^,^40^. Our estimation for the divergence time of *Hydnum* is about 67 Mya, with an apparent acceleration of speciation rate since about 30–40 Mya (Fig. 4), which could be attributed to the climatic fluctuation since the boundary of the Eocene and the Oligocene (about 34 Mya)^41^,^42^. The estimated time scale for the diversification of *Hydnum* is consistent with the existence of the North Atlantic Land Bridge (NALB) and the Bering Land Bridge (BLB), and also matches the migration time and routes of one putative ectomycorrhizal partner of *Hydnum*, Fagaceae, which has spanned its distribution range to Asia, Europe and North America during the Oligocene through the floristic exchanges earlier via the NALB and later via the BLB^43^. Within the continents of America, *Hydnum* species found in Central America and northern South America could be the result of recent migration from North America via the Panamanian Land Bridge, which was also found in the plant genus *Quercus*^44^. We could provide here *H. repandum* as an example. This species was shown in our study to be a holarctic species, which also extends its range into northern South America. An ITS sequence (JQ063050, from Venezuela) generated from root tips of the dipterocarp *Pakaraimaea dipterocarpacea,* a plant endemic to South America^45^, is nested within *H. repandum*. This sequence is identical to many sequences generated from European *H. repandum*, and shows very limited differences from the sequence generated from root tips of *Pinus muricata* (GU180269, from California) (Fig. 1). This suggests a recent migration followed by a host switch to *Pakaraimaea dipterocarpacea.*

Within the continent of Eurasia, six species exhibited an East Asian–European discontinuous distribution pattern (four with holarctic distribution). Interestingly, four species, viz. *H. magnorufescens*, *H. repandum*, *H. rufescens* and *Hydnum vesterholtii*, could be found from both northeastern/northern China (or Russia) and subalpine areas of the Hengduan Mountains region in southwestern China (Fig. 1). This is contrary to our finding on the porcini mushrooms, which has illustrated that fungi from northeastern/northern China would be phylogenetically closer to European ones, while those from southwestern China usually form unique lineages endemic to this region^25^. This kind of distribution pattern found in *Hydnum* strongly suggests that the composition of the mycobiota in the Hengduan Mountains reigion is more complicated than what we have known. Some of these species could be remnants of earlier diversified *Hydnum,* such as *H. repandum* and *H. vesterholtii,* as they show significant divergences among samples from southwestern and northeastern/northern China. Similar remnant species, *H. ellipsosporum*, could also be detected from the subalpine area of the Central Mountain Range in Taiwan, China (Fig. 1). In contrast, some species, like *H. magnorufescens* and *H. rufescens*, could have colonized the Hengduan Mountains region much later, as limited differentiations can be observed on samples from southwestern China, northeastern/northern China and Europe. In addition, the discontinuous distribution of *Hydnum* between Europe and southwestern China could, for the first time, infer the Tethys region as one of the possible corridors for the historical biota exchanges of ectomycorrhizal fungi between Europe and East Asia, which has been hypothesized by several biogeographical studies on different plant groups^46^.

To summarize, we reconstructed the phylogeny of worldwide *Hydnum* for the first time by using sequences of four gene markers. Our study revealed unexpected rich species diversity in this fungal genus, with 15 new phylogenetic species (nearly half of the known species) identified. A significant disjunct distribution pattern between the Palaeoarctic and the Neoarctic was detected, suggesting frequent historical biota exchanges via both the NALB and the BLB. Most species from Australasia and the subalpine areas of the Hengduan Mountains region in southwestern China were illustrated as old relics. Unfortunately, we failed to determine a center of origin for *Hydnum.* Further research with special focus on samples from Africa, South America and tropical Asia may help resolve this question.

## Materials and Methods

### Sample gathering

A broad survey for *Hydnum* has been made in southwestern, central, southern and northeastern China during the last ten years. Several collections were also made from Slovenia, the USA, Germany, Sweden, Russia, Australia, Malaysia and Singapore. These specimens are kept in the Cryptogamic Herbarium (HKAS) of Kunming Institute of Botany, Chinese Academy of Sciences, the herbarium at Slovenian Forestry Institute (LJU) and the herbarium of the Forest Research Institute Malaysia (FRIM). To understand this genus better in a worldwide view, specimens from northeastern China, Russia, the USA, Canada, Costa Rica, Germany, Australia and New Zealand were borrowed from several herbaria (HMJAU, F, PERTH and PDD) and included in our comparative analyses. Detailed information for the samples used in this study is listed in Supplementary Table S1.

### DNA extraction, fragment amplification and sequencing

Genomic DNA was isolated from silica-gel-dried samples or from herbarium specimens using the CTAB method^47^. Sequences of four DNA fragments were generated for phylogenetic analyses, including gene fragments coding for the largest subunit of RNA polymerase II (*rpb*1) and the translation elongation factor alpha (*tef*1α), as well as two non-protein coding regions within the nuclear ribosomal RNA gene cluster: ITS and the large subunit of nuclear ribosomal RNA (nrLSU). Primer pairs ITS5/ITS4^48^ and LR0R/LR7^49^ were used to amplify ITS and nrLSU, respectively. For the amplification of *rpb*1 and *tef*1α, a total of 13 new primers (Table 1) were designed using the online tool Primer 3^50^. PCR reactions were conducted on an ABI 2720 Thermal Cycler (Applied Biosystems, Foster City, CA, USA) or an Eppendorf Master Cycler (Eppendorf, Netheler-Hinz, Hamburg, Germany) and the reaction conditions were as follows: pre-denaturation at 94 °C for 3 min, then followed by 35 cycles of denaturation at 94 °C for 40 s, annealing at 48 °C (for ITS), 50 °C (for nrLSU) or 53 °C (for *rpb*1 and *tef*1α) for 40 s, elongation at 72 °C for 90 s, afterward, a final elongation at 72 °C for 8 min was included after the cycles. PCR products were purified with a Gel Extraction & PCR Purification Combo Kit (Spin-column) (Bioteke, Beijing, China) and then sequenced in both directions on an ABI-3730-XL DNA Analyzer (Applied Biosystems, Foster City, CA, USA) using the same primers as in the PCR amplifications. Direct sequencing of ITS for some specimens failed. We thus subcloned these DNA fragments into *Escherichia coli* strain DH5α using pMD18-T (Takara, Dalian, China) as a vector and then finished the sequencing by using the bacterial colony directly.

### Sequence alignments and phylogenetic analyses

The newly generated sequences were carefully checked with the chromatograms. Vector sequences were trimmed. All sequences were blasted over sequences available in GenBank to exclude possible contamination. As GenBank holds mainly ITS sequences for this genus, we firstly compiled a dataset of ITS sequences to investigate the species diversity of *Hydnum*. All ITS sequences of *Hydnum* available in GenBank and the UNITE databases were retrieved for comparative analyses, but the sequences with poor quality (with very short lengths or with too many degenerate bases) and redundant identical sequences in a same phylogenetic species were later excluded from our final analyses. Most Australasian samples showed significant ITS sequence divergences from samples from the Northern Hemisphere. It is thus impossible to unambiguously align the ITS sequences in a single matrix. Therefore, we divided the ITS sequences into two datasets: ITS dataset I including samples from the Northern Hemisphere and two accessions made from Papua New Guinea (UDB013289 and UDB013043) and ITS dataset II made up by sequences generated from the remaining Australasian samples. Besides the two ITS matrices, we built up three matrices consisting of sequences of nrLSU, *rpb*1 and *tef*1α, respectively, which were later concatenated together to form a three-gene matrix. For each single-gene dataset, sequences were aligned using mafft v7.127^51^ and then manually adjusted in 4SALE^52^. For the three-gene dataset, sequences of the three DNA fragments were concatenated with SequenceMatrix v1.7.8^53^.

We selected the best partition schemes for ITS dataset I, *rpb*1, *tef*1α and the three-gene matrices using PartitionFinder v1.1.1^54^. The best evolutionary model for each of the selected partitions was simultaneously estimated in the same software. Prior to partition scheme selection, the data blocks were manually defined. ITS dataset I was divided into three blocks, ITS1, 5.8 S and ITS2, nrLSU was treated as a single block, while *rpb*1 and *tef*1α were each separated into three blocks by the 1^st^, 2^nd^ and 3^rd^ codons. The best partition schemes for these datasets are summarized in Table 2.

Both ML and BI strategies were used to analyze all datasets (except for ITS dataset II) by using RaxML 8^55^ and MrBayes v3.2.3^56^, respectively. The “partitioned analysis” model was employed in the phylogenetic analyses of ITS dataset I, *rpb*1, *tef*1α and the three-gene matrices. In ML analyses, the GTRGAMMAI model was selected for all the partitions in different datasets, and the remaining parameters were kept as the default setting. Statistical support values were obtained by using nonparametric bootstrapping with 1000 replicates. BI analyses using selected models and four chains were conducted by setting generations to five million and using the “stoprul” command with the value of “stopval” set to 0.01. Trees were sampled every 100 generations. Chain convergence was determined using Tracer v1.5 (http://tree.bio.ed.ac.uk/software/tracer/) to ensure sufficiently large effective sampling size values (>200). Burn-ins were then determined by checking the 2lnL trace plots in Tracer. Subsequently, sampled trees were summarized with burn-ins discarded by using the “sumt” command implemented in MrBayes to get posterior possibilities. For ITS dataset II, we constructed a Neighbor-Joining (NJ) tree by using Mega 6.06^57^ to distinguish species, as the alignment showed some ambiguity, which makes it impossible to precisely infer the phylogenetic relationships among these species by using this dataset. All parameters were kept at their default values when constructing the NJ tree except that a bootstrap method was employed to test the phylogeny by setting the number of bootstrap replications to 1000.

### Phylogenetic species delineation

We adopted the Genealogical Concordance Phylogenetic Species Recognition^58^ to delimit phylogenetic species within *Hydnum*. A phylogenetic species was defined as a well-supported monophyletic clade which can fulfill either of the following two criteria^59^: (i) it is concordantly supported by the majority of the four loci; (ii) it is well supported by at least one locus but not significantly contradicted by any other locus.

### Divergence time estimation

Sequences of *rpb*1 and *tef*1α were used to estimate the divergence time of *Hydnum* by using BEAST v1.8.1^60^. Sequences of *rpb*1 and *tef*1α for *Clavulina* sp., *Botryobasidium botryosum* (Bres.) J. Erikss., *Calocera cornea* (Batsch) Fr., *Calocera viscosa* (Pers.) Fr., *Cryptococcus neoformans* (San Felice) Vuill., *Agaricostilbum hyphaenes* (Har. & Pat.) Oberw. & Bandon, *Schizosaccharomyces pombe* Lindner, *Morchella conica* Pers., and *Rhizopus microsporus* Tiegh. were retrieved from their genomes available in MycoCosm (http://jgi.doe.gov/fungi). We followed our previous studies on the porcini mushrooms^25^ to select the calibration point. Only exons were included in the analyses. The alignment and the concatenation of sequences, as well as the selection of the best partition scheme and evolutionary models, were the same as what were used in the phylogenetic analyses. The best partition scheme selected was as follows: the 1^st^ and 2^nd^ codons of *tef*1α were defined as one partition, with K80 + I + G as its best evolutionary model; the 3^rd^ codons of *tef*1α, and the 1^st^, 2^nd^ and 3^rd^ codons of *rpb*1 each represented a separate partition, with HKY + G, TrNef + I + G, SYM + I and HKY + I + G as their best evolutionary models, respectively. To evaluate different clock models, we followed the manual of BEAST. We firstly analyzed the data using a relaxed lognormal clock model and then checked the values of “ucld. stdev” parameters achieved. An “ucld.stdev” close to 0.0 supports that the data used are clock-like; otherwise, it rejects this hypothesis. We then compared the relaxed lognormal clock model and relaxed exponential clock model by running two separate analyses based on these two models respectively and then comparing the marginal likelihoods of the posteriors of different runs using the model comparison function available in Tracer v1.6. Other procedures for the estimation of divergence time were the same as those used in our previous study on the porcini mushrooms^25^.

## Additional Information

**How to cite this article**: Feng, B. *et al.* Multilocus phylogenetic analyses reveal unexpected abundant diversity and significant disjunct distribution pattern of the Hedgehog Mushrooms (*Hydnum* L.). *Sci. Rep.* **6**, 25586; doi: 10.1038/srep25586 (2016).

## Supplementary Material

Supplementary Information

## Figures and Tables

**Table 1:** Primers designed for the amplifications of *rpb*1 and *tef*1*α.*

DNA fragment	Primer	Sequence of primer
*rpb1*	H*rpb1*F (Forward)	5′-TGCAGTTCGTGCTCATTTTC-3′
	H*rpb1*R (Reverse)	5′-CGTGTCTACACGCTGTCGTT-3′
	H*rpb1*-1 (Forward)	5′-CAACAARCCTGTRATGGGWA-3′
	H*rpb1*-2 (Reverse)	5′-GCRGTRTCRATGAGACCYTC-3′
	H*rpb1*-3 (Forward)	5′-GCAGAGYGAGGAAACTCGAG-3′
	H*rpb1*-4 (Forward)	5′-AGCTTAGCCAAATCGCATGG-3′
	H*rpb1*-5 (Reverse)	5′-CGTTGRCCTTGTTCGATRTA-3′
*tef1*α	HEF1F (Forward)	5′-AATCTCTGGTTGGCATGGAG-3′
	HEF1R (Reverse)	5′-TTCCATCGTCTTTCCTGTCC-3′
	HEF1-1 (Forward)	5′-CATGTTGGAGGAATCWGTYAAG-3′
	HEF1-2 (Reverse)	5′-GGGGTGRTTAAGAACGATRA-3′
	HEF1-3 (Forward)	5′-GGCATGGAGACAACATGTTGGA-3′
	HEF1–4 (Reverse)	5′-TCCGACGATCRATCTTCTCG-3′

**Table 2:** The best partition scheme selected by PartitionFinder for each dataset used in phylogenetic analyses.

Dataset	MrBayes		RAxML	
	Partition scheme	Model	Partition scheme	Model
ITS	ITS1; ITS2	HKY + G	ITS1; ITS2	GTR + G
	5.8 S	K80 + I	5.8 S	GTR + I + G
nrLSU	full	HKY + I + G	Full	GTR + I + G
*rpb*1	codon 1	HKY + I	codon 1, 2	GTR + G
	codon 2	K80	codon 3	GTR + G
	codon 3	HKY		
*tef*1*α*	codon 1, 2	K80 + I + G	codon 1, 2	GTR + I + G
	condon 3	HKY + G	codon 3	GTR + G
3-gene	*rpb*1_codon 1; *tef*1*α*_codon 1	HKY + I + G	*rpb*1_codon 1, 2; *tef*1*α*_codon 1, 2	GTR + I + G
	*tef*1*α*_codon2	JC + I	*rpb*1_codon 3; *tef*1*α*_codon 3	GTR + G
	*rpb*1_codon 3; *tef*1*α*_codon 3	HKY + G	nrLSU	GTR + I + G
	nrLSU	HKY + I + G		
*rpb*1_codon2	K80			
