# Supplementary material for: Multilocus phylogenetic analyses reveal unexpected abundant diversity and significant disjunct distribution pattern of the Hedgehog Mushrooms (Hydnum L.)
Source: Sci Rep. 2016 May 6;6:25586. doi: 10.1038/srep25586 (PMC4858670; doi:10.1038/srep25586)
Supplement: Supplementary Information [file srep25586-s1.pdf]

Multilocus phylogenetic analyses reveal unexpected abundant diversity and significant disjunct distribution pattern of the Hedgehog Mushrooms (*Hydnum* L.)

Bang Feng[1] Xiang-Hua Wang[1] David Ratkowsky[2] Genevieve Gates[2] Su See Lee[3] Tine Grebenc[4] Zhu L. Yang[1*]

1 Key Laboratory for Plant Diversity and Biogeography of East Asia, Kunming Institute of Botany, Chinese Academy of Sciences, Kunming, China

2 Tasmanian Institute of Agriculture, and School of Plant Science, University of Tasmania, Hobart, Tasmania, Australia

3 Tanarimba, Janda Baik, Bentong 28750 Bentong, Pahang, Malaysia

4 Slovenian Forestry Institute, Večna pot 2, SI-1000 Ljubljana, Slovenia

*Correspondence: Zhu L. Yang, Key Laboratory for Plant Diversity and Biogeography of East Asia, Kunming Institute of Botany, Chinese Academy of Sciences, Kunming 650201, Yunnan, China, fungi@mail.kib.ac.cn

Table S1 Specimens used for phylogenetic analyses and the GenBank accessions for the sequences generated in this study.

**Taxon**	**Collection ID**	**Herbarium ID**	**Location**	**GenBank Accessions**			
				**ITS**	**nrLSU**	***rpb*1**	***tef*1*α***
H. crocidens	—	PDD93278	New Zealand: Auckland, Hunua, Workman Road, Workman Track	KU612632			
H. crocidens	—	PERTH08072965	Australia: About 100m south of Rosa Brook, near Marganet River	KU612630	KU612685		
H. crocidens	—	PERTH08095981	Australia: Augusta, west of Bussell Highway, Donovan Bushland	KU612631	KU612684		KU612797
H. elatum	—	FRI61322	Malaysia: Trail to Tower, Huran Simpan Keal Pasoh	KU612639			
H. elatum	—	FRI61713	Malaysia: Tasik Bera Pahang-Taman Herba	KU612634			
H. elatum	—	FRI62150	Malaysia: Hutan Ramsah, Felda Selatan 3	KU612638	KU612692		KU612812
H. elatum	—	FRI62309	Malaysia: Kampung Jelawat-Tasik Bera Pahang	KU612637	KU612691	KU612755	KU612811
H. elatum	—	FRI62814	Malaysia : H/S, Pasoh Nature Trail	KU612636	KU612693		
H. elatum	Hao Yang	HKAS92352	Singapore	KU612633		KU612756	KU612810
H. elatum	—	FRI62317	Malaysia: Tasik Bera Pahang-Taman Herba	KU612635	KU612694		
H. ellipsosporum	—	HMJAU5985	China: Jilin Province, Antu County, Changbai Mountain	KU612602			
H. ellipsosporum	Yang M22	HKAS93254	Germany	KU612604		KU612726	KU612791
H. ellipsosporum	Yang M31	HKAS93255	Germany	KU612605			
H. ellipsosporum	Yang4993	HKAS56491	Germany	KU612603			
H. magnorufescens	—	HMJAU4677	Russia: Kyrov, Belaya Holunitsa	KU612550			
H. magnorufescens	161209	—	Slovenia	KU612549	KU612669	KU612700	KU612795
H. ovoideisporum	071106	—	Slovenia	KU612536		KU612711	
H. repandum	KR9177	—	Germany	KU612575			
H. repandum	Yang M21	HKAS93253	Germany	KU612581		KU612731	KU612769
H. repandum	161209B	—	Slovenia	KU612580			
H. repandum	161209A	—	Slovenia	KU612578			
H. repandum	231109B	—	Slovenia	KU612577			
H. repandum	251209	—	Slovenia	KU612579			

H. repandum	031209C	—	Slovenia	KU612576			
H. repandum	031209A	—	Slovenia	KU612574	KU612655	KU612732	KU612770
H. repandum	Yang5120	HKAS54416	China: Jilin Province, Antu County, Changbai Mountain	KU612583			
H. repandum	Wang3596	HKAS92333	Sweden: Oestersund, Sandviken, Fillstabaecken Nature Reserve	KU612582			
H. rufescens	Li1388	HKAS56228	China: Jilin Province	KU612540			
H. rufescens	Feng609	HKAS57338	China: Tibet, Jiangda county, Guopubaizong Mountain	KU612542			
H. rufescens	Shi585	HKAS92339	China: Heilongjiang Province, Yichun County, Tangwang River Nature Reserve	KU612537	KU612658	KU612712	KU612783
H. rufescens	Shi468	HKAS92338	China: Jilin Province, Antu County, Changbai Mountain	KU612538	KU612659	KU612713	KU612784
H. rufescens	Feng1423	HKAS82529	China: Tibet Autonomous Region, Changdu County, Lamei	KU612541	KU612657	KU612714	KU612781
H. rufescens	Wang3598	HKAS92337	Sweden: Oestersund, Sandviken, Fillstabaecken Nature Reserve	KU612539	KU612656	KU612725	KU612780
H. rufescens	Feng1433	HKAS82539	China: Sichuan Province, Dege County, Manigange			KU612715	KU612782
H. sp.	Feng656	HKAS57385	China: Yunnan Province, Shangeri-La County, Bita Lake	KU612601			
H. sp.	—	F1110834	Costa Rica: Cartago, Estrella	KU612598			
H. sp.	—	F1104787	USA: Michigan, Manistee County	KU612606			
H. sp.	Wang2369	HKAS61337	China: Hunan Province, Zhangjiajie	KU612597	KU612644	KU612699	KU612759
H. sp.	—	FRI62832	Malaysia: H/S Pasoh-Denai Alam	KU612625			
H. sp.	GD1586	—	Australia: Tasmania, Zig-Zag Track			KU612750	KU612807
H. sp.	Feng1269	HKAS82411	China: Taiwan, Nantu County, Hohuan Mountain	KU612607	KU612668	KU612728	KU612793
H. sp. 1	231109	—	Slovenia	KU612545	KU612660	KU612716	KU612794
H. sp. 2	G. Michalenko	F1187537	Canada: Alberta, Jasper Park County	KU612544			
H. sp. 2	Shi613	HKAS92340	China: Heilongjiang Province, Daxing'anling	KU612543	KU612661	KU612717	KU612779
H. sp. 3	Li1274	HKAS56128	China: Yunnan Province, Yulong County	KU612526			
H. sp. 3	Tang924	HKAS56881	China: Yunnan Province, Changning County	KU612530			
H. sp. 3	Wu158	HKAS57690	China: Yunnan Province	KU612527			
H. sp. 3	Feng693	HKAS57422	China: Yunnan Province, Yulong County	KU612529			

H. sp. 3	Feng746	HKAS57475	China: Yunnan Province, Jianchuan County	KU612528	KU612666	KU612722	KU612796
H. sp. 3	Wang2594	HKAS61795	China: Heilongjiang Province, Mulan County, Youyi	KU612531	KU612665	KU612718	KU612776
H. sp. 3	Feng1090	HKAS74570	China: Yunnan Province, Tengchong County, Houqiao	KU612522			
H. sp. 3	Feng1121	HKAS74602	China: Yunnan Province, Baoshan, Longyang	KU612532			
H. sp. 3	Feng1123	HKAS74604	China: Yunnan Province, Baoshan, Longyang	KU612533			
H. sp. 3	Feng1162	HKAS74643	China: Yunnan Province, Lanping County, Hexi	KU612523			
H. sp. 3	Qin433	HKAS77834	China: Hunan Province, Shunhuangshan Natural Forest Park	KU612525	KU612667	KU612721	
H. sp. 3	Feng1375	HKAS93262	China: Yunnan Province, Jingdong County, Ailao Mountain	KU612524		KU612724	
H. sp. 3	Fan YG-1	HKAS92348	China: Jilin Province		KU612664	KU612719	KU612777
H. sp. 3	Fan YG-2	HKAS93256	China: Jilin Province			KU612720	KU612778
H. sp. 4	Siyi Li 31	F1125199	USA: Wisconsin, Walworth County, LaGrange east loop	KU612534			
H. sp. 4	EK Karczynski 42	F1188749	USA: Illinois, Cook County	KU612535	KU612663	KU612723	KU612785
H. sp. 6	NAMA 2008-011	F1186911	USA: Idaho, Adams County, McCall Hall Ranch	KU612548	KU612662	KU612703	KU612775
H. sp. 6	Yang4390	HKAS45769	China: Tibet Autonomous Region, Leiwuqi County, Binda	KU612547		KU612701	KU612773
H. sp. 6	Feng1402	HKAS82508	China: Tibet Autonomous Region, Mangkang County, Rumei	KU612546	KU612670	KU612702	KU612774
H. sp. 7	Feng209	HKAS55319	China: Yunnan Province, Mengla County, Menglun	KU612590			
H. sp. 7	Feng210	HKAS55320	China: Yunnan Province, Mengla County, Menglun	KU612591			
H. sp. 7	Zeng158	HKAS93257	China: Yunnan Province	KU612588			
H. sp. 7	Feng11	HKAS51070	China: Yunnan Province, Jingdong County, Ailao Mountain	KU612584			
H. sp. 7	Feng12	HKAS51071	China: Yunnan Province, Jingdong County, Ailao Mountain	KU612585			
H. sp. 7	Feng13	HKAS51072	China: Yunnan Province, Jingdong County, Ailao Mountain	KU612586			
H. sp. 7	Feng29	HKAS51088	China: Yunnan Province, Kunming, Qiongzhu Temple	KU612587			
H. sp. 7	Li Fang 1177	HKAS78334	China: Guangdong Province, Fengkai County, Heishiding	KU612589		KU612733	KU612768

H. sp. 8	Feng299	HKAS55410	China: Yunnan Province, Shangeri-La County, Haba Snow Mountain	KU612596	KU612654	KU612729	KU612771
H. sp. 8	Feng338	HKAS55449	China: Yunnan Province, Shangeri-La County, Daxue Mountain	KU612594			
H. sp. 8	Wu235	HKAS57767	China: Yunnan Province, Yulong County, Laojun Mountain	KU612592			
H. sp. 8	Feng783	HKAS68574	China: Yunnan Province, Yulong County, Laojun Mountain	KU612593			
H. sp. 8	Feng1452	HKAS82558	China: Sichuan Province, Hongyuan County	KU612595		KU612730	KU612772
H. sp. 9	ER Ewald 67	F1188765	USA: Indiana, Porter County	KU612599	KU612653	KU612741	
H. sp. 10	Feng217	HKAS55327	China: Yunnan Province, Jingdong County, Ailao Mountain	KU612568	KU612682	KU612737	
H. sp. 10	Zhao185	HKAS93260	China: Yunnan Province	KU612572			
H. sp. 10	Tang829	HKAS56789	China: Yunnan Province, Yingjiang County, Xima	KU612571	KU612680	KU612736	KU612766
H. sp. 10	Tang941	HKAS56898	China: Yunnan Province, Changning County	KU612570			
H. sp. 10	Feng1062	HKAS74541	China: Yunnan Province, Tengchong County, Houqiao	KU612566			
H. sp. 10	Feng1268	HKAS82410	China: Taiwan, Nantu County, Hohuan Mountain	KU612573	KU612679	KU612734	KU612765
H. sp. 10	Feng1365	HKAS93261	China: Yunnan Province, Jingdong County, Ailao Mountain	KU612567	KU612681	KU612735	KU612767
H. sp. 10	Wang3461	HKAS92334	China: Yunnan Province, on the way from Ninger to Puer	KU612569			
H. sp. 11	—	F1185236	USA: West Virginia, Raleigh County, Beaver	KU612600			
H. sp. 12	Zeng452	HKAS93258	China: Hainan	KU612618	KU612677	KU612743	KU612763
H. sp. 12	XB-1	HKAS92345	China: Chongqing, Nanchuan County, Jinfo Mountain	KU612619	KU612676	KU612742	KU612764
H. sp. 13	Wu182	HKAS57714	China: Yunnan Province, Jianchuan County	KU612617	KU612673	KU612740	KU612762
H. sp. 13	Cai173	HKAS58838	China: Yunnan Province, Jianchuan County	KU612616	KU612675	KU612738	KU612760
H. sp. 13	Feng752	HKAS57481	China: Yunnan Province, Yunlong County, Shitou	KU612615			
H. sp. 13	XB-2	HKAS92346	China: Chongqing, Nanchuan County, Jinfo Mountain	KU612560	KU612674	KU612739	KU612761
H. sp. 15	Feng215	HKAS55325	China: Yunnan Province, Jingdong County, Ailao Mountain	KU612613			
H. sp. 15	Zhao131	HKAS93259	China: Yunnan Province, Mengla County	KU612612	KU612683	KU612745	KU612803
H. sp. 15	Cai9	HKAS58676	China: Yunnan Province	KU612611			

H. sp. 15	Wang3503	HKAS92336	China: Yunnan Province, Simao District, Caiyanghe Natural Reserve	KU612614		KU612744	KU612802
H. sp. 16	Feng87	HKAS52807	China: Yunnan Province, Yunlong County, Laojun Mountain	KU612609		KU612748	KU612801
H. sp. 16	GY3559	HKAS92350	China: Guizhou Province	KU612610	KU612672	KU612747	KU612799
H. sp. 16	XB-3	HKAS92347	China: Chongqing, Nanchuan County, Jinfo Mountain		KU612652	KU612749	KU612800
H. sp. 16	GY153	HKAS92349	China: Guizhou Province		KU612671	KU612746	KU612798
H. sp. 17	—	PDD93275	New Zealand: Auckland, Hunua, Workman Road, Workman Track	KU612624			KU612806
H. sp. 17	—	PDD98029	New Zealand: Wairarapa Tararua Forest Park, Mt. Holdsworth	KU612623	KU612690		
H. sp. 17	—	PERTH07830742	Australia: Denmark, William Bay National Park	KU612622	KU612689		
H. sp. 17	—	PERTH08072957	Australia: About 100m south of Rosa Brook, near Marganet River	KU612621			
H. sp. 17	GD1588	—	Australia: Tasmania, Pipeline track,Mt. Wellington	KU612620	KU612688	KU612752	KU612805
H. sp. 18	—	PDD94968	New Zealand: Dunedin, Leith Valley, Swampy Spur	KU612627			
H. sp. 18	GD1590	—	Australia: Tasmania, Shinglebend, Styx River, Styx Valley	KU612629	KU612686	KU612754	KU612809
H. sp. 18	GD1589	—	Australia: Tasmania, Shinglebend, Styx River, Styx Valley	KU612628	KU612687	KU612753	KU612808
H. sp. 19	—	PERTH07608543	Australia: Dwellingup, Amphion Forest Block, Murray River Road	KU612641			
H. sp. 19	—	PERTH08018413	Australia: Denmark, Loc 406, 8km west of Denmark	KU612642	KU612696		
H. sp. 19	—	PERTH08091676	Australia: Denmark, Mt. Romance Farm, Tindale Road	KU612640			
H. sp. 19	—	PERTH08093865	Australia: Augusta, West Bay bushland, Leeuwin-Naturaliste National Park	KU612643	KU612695	KU612751	KU612804
H. umbilicatum	Wang3312	HKAS92335	USA: Califonia, Mendocino	KU612608	KU612678	KU612727	KU612792
H. vesterholtii	Li1359	HKAS56213	China: Yunnan Province, Yulong County, Yulong Snow Mountain	KU612554			
H. vesterholtii	Feng194	HKAS52915	China: Yunnan Province, Shangeri-La County, Haba Snow Mountain	KU612553			
H. vesterholtii	Feng288	HKAS55399	China: Yunnan Province, Yulong County, Yulong Snow Mountain	KU612555			
H. vesterholtii	Feng290	HKAS55401	China: Yunnan Province, Yulong County, Yulong Snow Mountain	KU612552			
H. vesterholtii	Tang1090	HKAS57047	China: Yunnan Province, Nanhua County, Maan Mountain	KU612558			

H. vesterholtii	Cai67	HKAS58734	China: Yunnan Province, Yongping County	KU612559			
H. vesterholtii	Feng27	HKAS51086	China: Yunnan Province, Shangeri-La County, Hongshan	KU612557	KU612650	KU612707	
H. vesterholtii	Shi656	HKAS92341	China: Shaanxi Province, Mei County, Yingtou	KU612562	KU612647	KU612709	KU612790
H. vesterholtii	Shi688	HKAS92342	China: Yunnan Province, Yulong County, Yulong Snow Mountain	KU612564	KU612646	KU612705	KU612786
H. vesterholtii	Shi685	HKAS92343	China: Sichuan Province, Puge County, Luoji Mountain	KU612563	KU612648	KU612710	
H. vesterholtii	Shi645	HKAS92344	China: Heilongjiang Province, on the way from Jiagedaqi to Songling	KU612556	KU612649	KU612706	KU612788
H. vesterholtii	Feng961	HKAS74441	China: Yunnan Province, Gongshan County, Bingzhongluo	KU612551			
H. vesterholtii	Qin483	HKAS77884	China: Hubei Province, Yichang, Shennongjia	KU612565	KU612645	KU612704	KU612787
H. vesterholtii	Yang4991	HKAS56489	Germany	KU612561			
H. vesterholtii	GY8125	HKAS92351	China: Guizhou Province		KU612651	KU612708	KU612789
S. aff. *subconfluens*	Feng127	HKAS52848	China: Yunnan Province, Shangeri-La County, Geza		KU612697	KU612757	KU612813
S. aff. *subconfluens*	Feng197	HKAS52918	China: Yunnan Province, Shangeri-La County, Haba Snow Mountain		KU612698	KU612758	KU612814

Abbreviations: *H.* = *Hydnum*; *S.* = *Sistotrema*

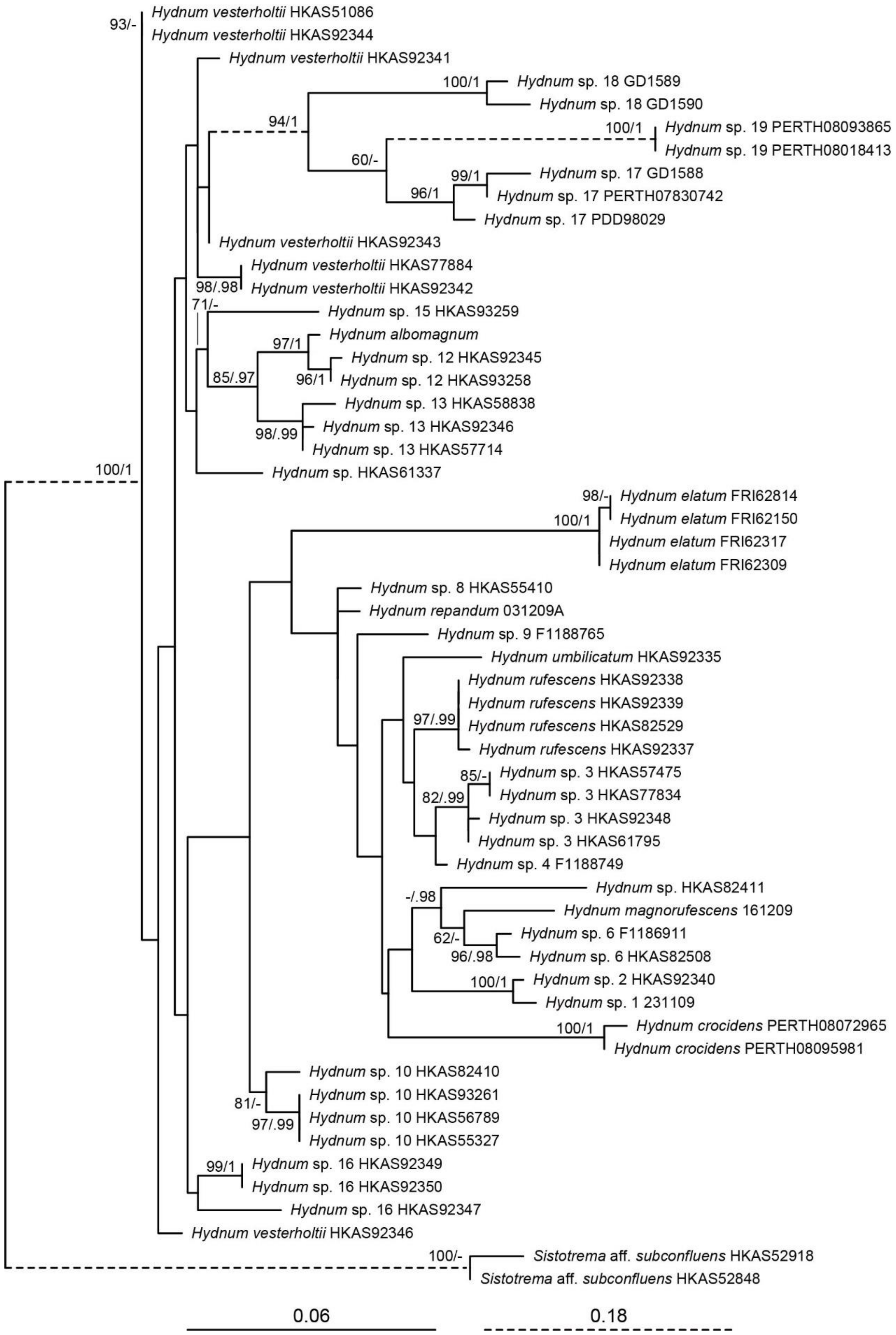

Fig. S1 Phylogenetic tree inferred from the Maximum Likelihood (ML) analysis based on the nrLSU dataset. Bootstrap values (ML)/posterior possibilities (from Bayesian Inference) are shown above or beneath individual branches. Only bootstrap values larger than 60 and posterior probabilities over 0.95 are shown.

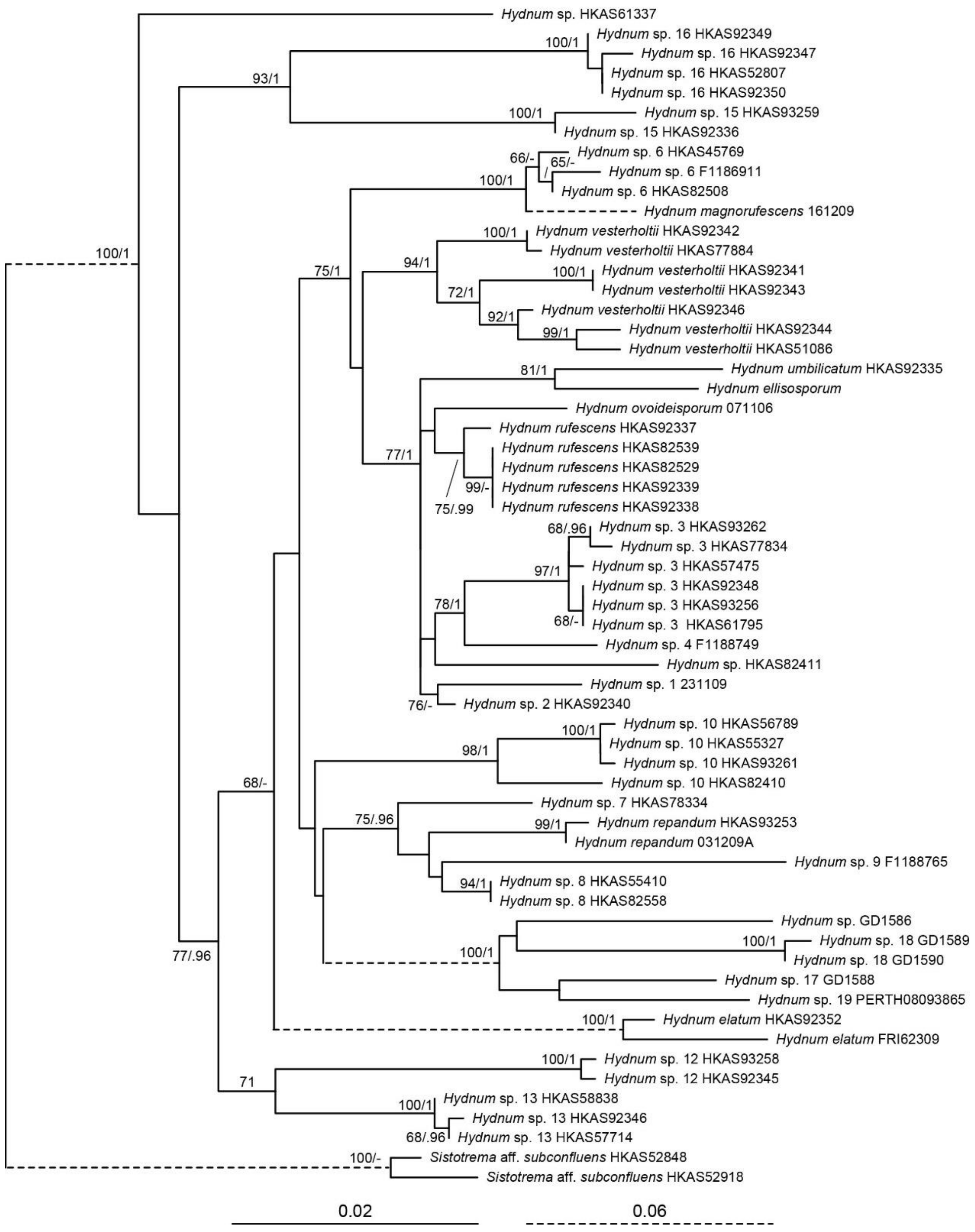

Fig. S2 Phylogenetic tree inferred from the Maximum Likelihood (ML) analysis based on the *rpb*1 dataset. Bootstrap values (ML)/posterior possibilities (from Bayesian Inference) are shown above or beneath individual branches. Only bootstrap values larger than 60 and posterior probabilities over 0.95 are shown.

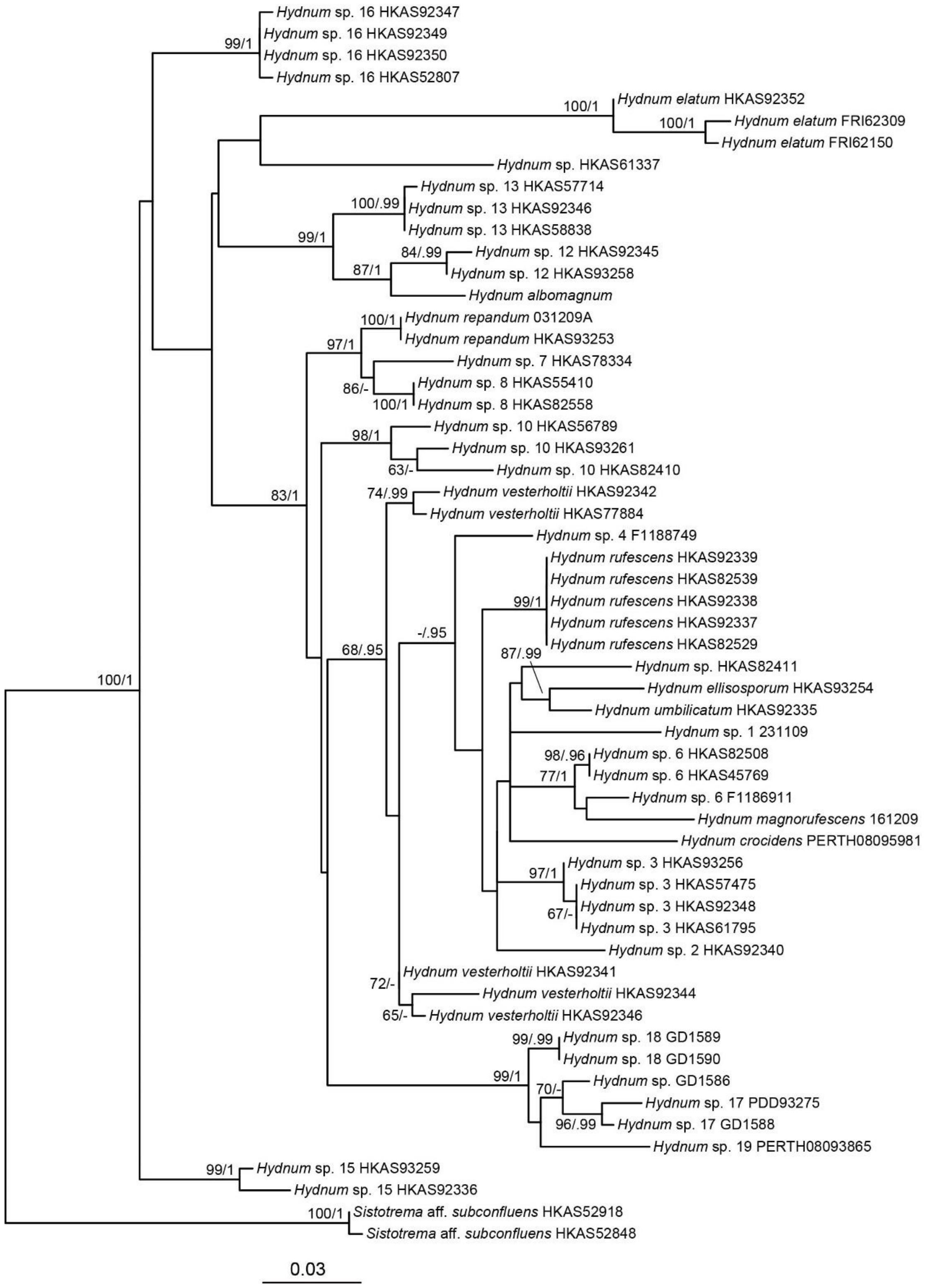

Fig. S3 Phylogenetic tree inferred from the Maximum Likelihood (ML) analysis based on the *tef*1α dataset. Bootstrap values (ML)/posterior possibilities (from Bayesian Inference) are shown above or beneath individual branches. Only bootstrap values larger than 60 and posterior probabilities over 0.95 are shown.
